# EXFI: Exon and splice graph prediction without a reference genome

**DOI:** 10.1002/ece3.6587

**Published:** 2020-07-28

**Authors:** Jorge Langa, Andone Estonba, Darrell Conklin

**Affiliations:** ^1^ Department of Genetics, Physical Anthropology and Animal Physiology Faculty of Science and Technology University of the Basque Country Leioa Spain; ^2^ Department of Computer Science and Artificial Intelligence, Faculty of Computer Science University of the Basque Country UPV/EHU San Sebastián Spain; ^3^ IKERBASQUE, Basque Foundation for Science Bilbao Spain

**Keywords:** exome sequencing, exon, sequence capture, SNP discovery, splice graph, transcriptome

## Abstract

For population genetic studies in nonmodel organisms, it is important to use every single source of genomic information. This paper presents EXFI, a Python pipeline that predicts the splice graph and exon sequences using an assembled transcriptome and raw whole‐genome sequencing reads. The main algorithm uses Bloom filters to remove reads that are not part of the transcriptome, to predict the intron–exon boundaries, to then proceed to call exons from the assembly, and to generate the underlying splice graph. The results are returned in GFA1 format, which encodes both the predicted exon sequences and how they are connected to form transcripts. EXFI is written in Python, tested on Linux platforms, and the source code is available under the MIT License at https://github.com/jlanga/exfi.

## Graphical abstract

EXFI predicts the splice graph from a transcriptome and selected WGS reads. Transcripts are splitted into exons given the information present in the WGS experiment. Predictions are suitable for downstream bioinformatic analyses and new experimental designs.

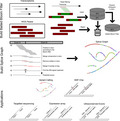

## 1 INTRODUCTION

In the last decade, high‐throughput sequencing technologies have enabled biologists to unravel the genetic code on a massive scale and at an unprecedented rate. However, sequencing and assembling whole genomes of nonmodel species is still not practical. Therefore, alternative approaches are needed to capture genetic variation. One approach commonly used in the context of population genetics is restriction site‐associated DNA sequencing (RAD‐Seq; Baird et al., 2008), which returns polymorphic markers at random loci across the entire genome. Posterior enhancements, such as RAD‐Seq followed by sequence capture (Rapture; Ali et al., 2016), have been recently proposed as an efficient and cost‐effective approach for genotyping thousands of samples and loci simultaneously (Meek & Larson, 2019).

**FIGURE 1:**
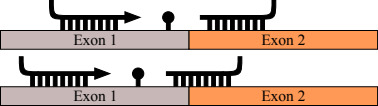
Two cases in primer design that can lead to genotyping failure: primers in different exons that require excessive PCR extension across an intron (top); a primer spanning an IEB will fail to anneal (bottom)

Another successfully proven and cost‐effective approach is to discover SNPs by sequencing both DNA and RNA and subsequently genotype large numbers of individuals (Kumar et al., 2019; Lamichhaney et al., 2012; Montes et al., 2013, 2015; Therkildsen & Palumbi, 2017). For these methods, attention is explicitly restricted to *transcriptomic SNPs*: Those contained inside expressed genes due to their higher functional relevance, rather than intergenic and intronic regions. The combined approach of DNA and RNA sequences to SNP discovery has obtained the highest nonmodel SNP validation rates to date, without requiring a reference genome, and its success is largely due to the accurate detection of intron–exon boundaries (IEBs), which can confound genotyping primer design (Wang et al., 2008; see Figure 1). The IEB detection method developed by Conklin, Montes, Albaina, and Estonba (2013), for example, relies on computing statistically significant positions in the transcript where many genomic reads start or end, indicating possible IEBs.

Traditional approaches to gene annotation in general, and IEB detection in particular, are based on the annotation of a genome assembly. For example, the NCBI Prokaryotic Genome Annotation Process (https://www.ncbi.nlm.nih.gov/genome/annotation_prok/process/, last accessed 2020‐03‐04) relies on the prediction of transcribable regions based on alignments to known transcripts and proteins, and ab initio predictors of coding and noncoding genes. A more popular solution is to align either transcriptome or RNA‐Seq reads with a splice‐aware aligner such as GMAP (Wu & Watanabe, 2005), and extract from the results the IEB coordinates.

An alternative approach to finding IEBs can be based on creating a splice graph, a mathematical representation of a transcriptome where exons are represented by nodes, IEBs as edges, transcripts as paths, and genes as the different connected components. This approach is the one first presented by ChopStitch (Khan et al., 2018) where Bloom filters are used to store frequent *k*‐mers of a shotgun whole‐genome sequencing (WGS) dataset and use it to find signals of splicing in every sequence of a transcriptome assembly.

This paper presents EXFI, a memory‐efficient tool for predicting and annotating the exons of a de novo transcriptome assembly through a splice graph representation. This tool works by comparing transcriptomic *k*‐mers with those sequenced in a WGS experiment, marking potential IEBs wherever a section of a transcript is not found in it. To assess its performance, we compare it with ChopStitch and GMAP, using two synthetic datasets where references are available (human being and zebrafish); two fish species (Atlantic herring and Atlantic salmon) for which there exist reference annotations and experimental WGS datasets; and two species for which there only a draft genome and transcriptome are available (sugar pine and axolotl). Finally, we applied EXFI to a recently published dataset on tench (Kumar et al., 2019) to evaluate its success in IEB detection for SNP discovery in exonic regions.

We expect this method to be useful not only in the context of the original aim, the decomposition of transcripts into exons for gene‐targeted SNP genotyping in organisms where genomic references are not available or not reliable, but also in the design of array‐based tools such as sequence and exome capture, exome‐wide genotyping, and RNA expression microarrays. Finally, recent developments in selective nanopore sequencing (Payne et al., 2020) are very likely to increase the relevance of exome‐targeted approaches such as the one described here.

## 2 METHODS

EXFI's core programs are written in Python, working on top of data processing (Pandas; McKinney, 2010) and Bioinformatics (BioPython; Cock et al., 2009) packages, as well as highly performant tools for *k*‐mer manipulation (BioBloomTools' commit 0a42916, Chu et al., 2014; ABySS 2.0.2, Jackman et al., 2017; BEDTools 2.27.1, Quinlan & Hall, 2010). Its three main programs are build_baited_bloom_filter, build_splice_graph, and gfa1_to_fasta, to create the underlying data structure, to predict the splice graphs, and to write the exons, respectively.

### 2.1 EXFI workflow

#### 2.1.1 Input

EXFI requires two input datasets: WGS reads and an assembled transcriptome in FASTQ and FASTA format, respectively. Such WGS reads may come from a single individual to multiple barcoded samples, even Pool‐Seq approaches. The transcriptome assembly can be a published reference (Ensembl or NCBI Genomes, for example), or a de novo result from short‐read or long‐read sequencing technologies.

#### 2.1.2 Baited bloom filter construction

A Bloom filter (BF; Bloom, 1970) is a fast and succinct data structure for set membership (i.e., to test whether a *k*‐mer is present in a transcript). Bloom filters have been successfully used in many high‐throughput sequencing problems, including *k*‐mer counting (Melsted & Pritchard, 2011), read compression (Benoit et al., 2015), read normalization (Crusoe et al., 2015), read filtering (Chu et al., 2014), error correction (Benoit, Lavenier, Lemaitre, & Rizk, 2014; Salmela & Rivals, 2014; Salmela, Walve, Rivals, & Ukkonen, 2017; Song, Florea, & Langmead, 2014), genome assembly (Chikhi, Limasset, & Medvedev, 2016; Chikhi & Rizk, 2012; Jackman et al., 2017; Peterlongo & Chikhi, 2012), gap filling (Paulino et al., 2015; Rizk, Gouin, Chikhi, & Lemaitre, 2014; Vandervalk et al., 2015), and targeted gene assembly (Kucuk et al., 2017). The advantage of this data structure is that it is very fast and space‐efficient, with the drawback of being probabilistic: It does not return false negatives, but it can produce false positives with a tunable false‐positive rate (BF FPR). This rate, for a given dataset, depends on three parameters that are under our control: the *k*‐mer length, the amount of memory, and the number of hash functions used.

In the human and zebrafish genomes, only 4.24% and 5.68% of the bases are exons, respectively (Table 1). Therefore, this Bloom filter approach can be used to remove WGS reads that are not exonic, and then reduce the BF FPR by nearly an order of magnitude. Additionally, cascading Bloom filters (Salikhov, Sacomoto, & Kucherov, 2014), a modification of original the data structure, stacks together multiple Bloom filters to keep frequent‐enough *k*‐mers and discard the ones produced by sequencing errors. Together, both approaches serve to filter out irrelevant but significant fractions of the original WGS experiment.

**TABLE 1:** Experimental statistics of the studied cases

Experiment	Zebrafish	Human being	Atl. salmon	Atl. herring	Sugar pine	Axolotl	Tench
Genome type	Chromosome	Chromosome	Chromosome	Scaffold	Scaffold	Chromosome	Not available
Genome size (Gbp)	1.34	3.09	2.97	0.81	27.60	32.40	0.78
Genes	25,497	21,407	79,030	25,135	Unknown	Unknown	Unknown
Transcriptome type	Reference	Reference	Reference	Reference/de novo	De novo	De novo	De novo
Transcripts	51,714	164,776	109,584	29,353/97,777	331,11	180,605	267,058
Transcriptome size (Mbp)	110.69	270.48	355.21	64.18/55.39	36.74	229.48	294.70
Exons	495,200	1,199,596	1,313,909	314,220/Unknown	Unknown	Unknown	Unknown
Samples	2	6	20	50	1	1	10
Reads (M)	720.00	2,160.00	1,259.27	418.73	9,300.90	7,121.91	318.72
Total bases (Gbp)	72.00	216.00	125.93	41.13	1,395.13	712.19	31.87
Coverage	53.73	69.90	42.44	50.92	50.54	21.98	51.58

Note

Genome sizes are the number of characters in their corresponding reference files. All species are diploid.

In EXFI, build_baited_bloom_filter uses both the transcriptome assembly and the WGS reads and performs the task in three steps. First, a Bloom filter of the transcriptome is built with biobloommaker. Second, each read of the WGS dataset that does not share at least one *k*‐mer with the transcriptome is discarded with biobloomcategorizer. And third, the remaining reads are used to build a cascading Bloom filter with ABySS. The result is a binary file encoding the error‐free *k*‐mers of the reads that overlap the transcriptome.

#### 2.1.3 Exon and splice graph prediction

The exon and splice graph prediction procedure is carried out by the build_splice_graph script, which predicts in one step the exon sequences, the exon composition of each transcript, and the splice graph structure of the entire transcriptome.

**FIGURE 2:**
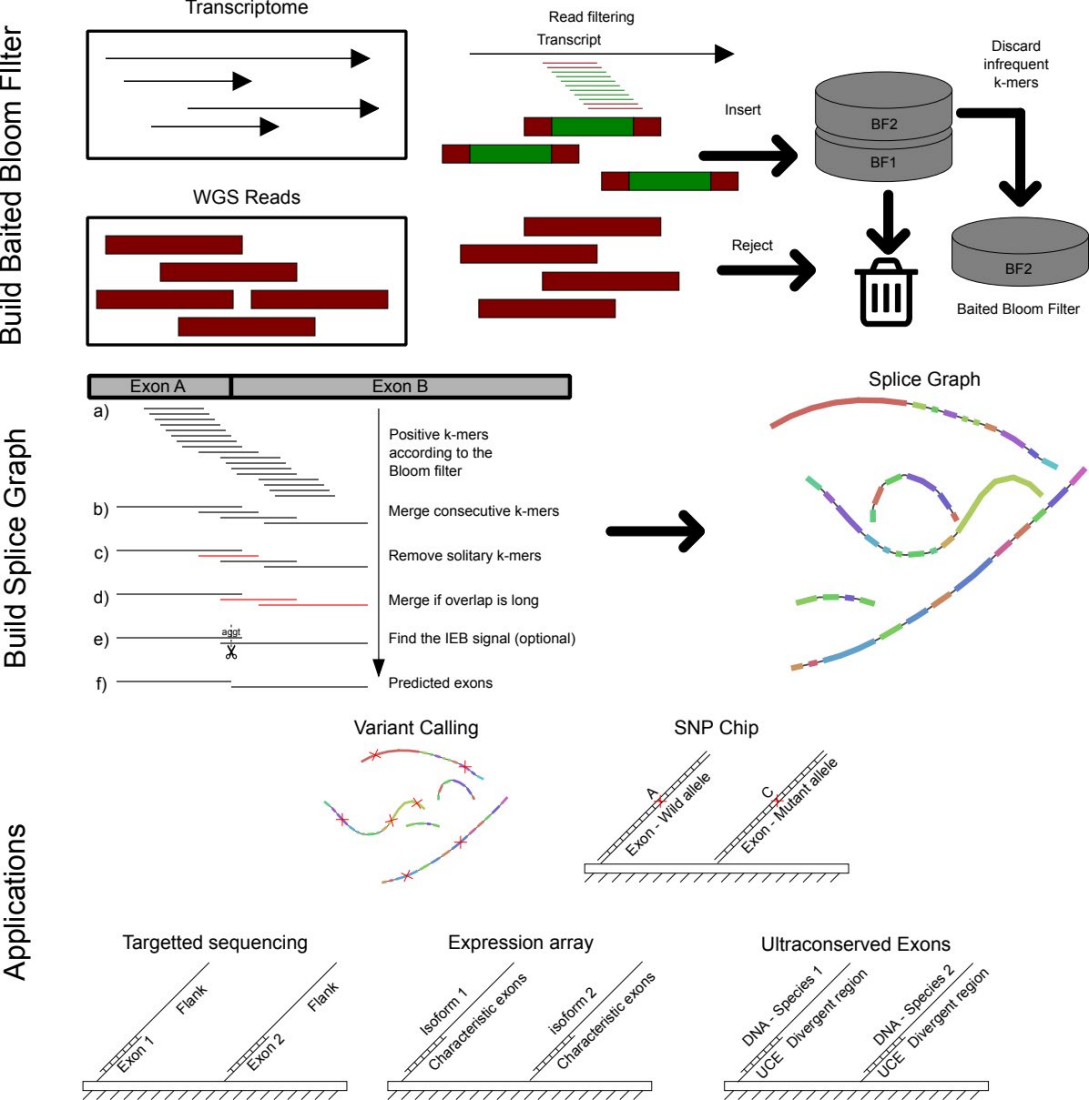
Schematic representation of the computational procedure. In the building stage, a WGS reads are filtered out according to whether or not they share a *k*‐mer with the transcriptome. Positive reads are inserted into the cascading Bloom filter. Only the last one is used for analysis. The prediction step is comprised of multiples steps in which: (a) every transcriptomic *k*‐mer is queried one by one to the filtered WGS set. (b) *k*‐mers overlap by *k* − 1 bases are merged together. (c) Exons that are likely to be false positives are thrown away by a minimum length criterion. d) Exons that overlap by too many bases (ten by default) are merged together. (e) Overlaps between pairs of exons are inspected to see whether it contains the AG‐GT splicing signal. (f) Exons are reported. Potential applications of EXFI include exome variant calling, design of SNP chips, targetted sequence of the exome, expression arrays, and UCE assays

First, transcriptomic *k*‐mers are inspected sequentially: Those that overlap two different exons should not be present in the WGS dataset (Figure 2a) and therefore mark where an exon ends and the following starts. Then, consecutive positive *k*‐mers that overlap by *k* − 1 bases are merged together, providing a draft exome (Figure 2b). The false positives that the Bloom filter produces may cause additional nucleotides in the raw exome and disconnected exons of length *k*. To prevent downstream problems, exons of length less than *k* + *q* (*q* by default five) are filtered out (Figure 2c). Once deleted, a more relaxed merging step is applied when exons overlap by an excessive number of bases (10 by default; Figure 2d). Finally, if the ‐polish flag is specified, each pair of exons with a long‐enough overlap is inspected for the donor/acceptor sites (usually GU/AG; 2e) and correctly trimmed if possible.

The primary output is a GFA1 file that encodes the inferred exons in terms of sequence and coordinates, the connections between them, and the transcripts as paths of exons. This type of file can be visualized with Bandage (Wick, Schultz, Zobel, & Holt, 2015), which also is helpful to manipulate exons and transcripts of interest, as well as to perform BLAST queries. Additionally, (gfa1_to_fasta) extracts the exons in FASTA format. It can also return the spliced transcripts, where each one of them is represented by the corresponding exons separated by a predefined amount of Ns.

### 2.2 Validation datasets

Four reference datasets were selected: zebrafish (*Danio rerio*) and human being (*Homo sapiens*) as the key species, due to the depth of their available annotations; and Atlantic herring (*Clupea harengus*) and Atlantic salmon (*Salmo salar*), both with complete assemblies and exon annotations. Also, *Salmoniformes* are known to have an additional genome duplication round not shared by the other fish species here studied (Allendorf & Thorgaard, 1984), expanding both the genome length and number of genes (and therefore transcriptome complexity; Table 1). Additionally, to serve as a bridge between reference and de novo transcriptome assemblies, an RNA‐Seq muscle library from Atlantic herring was assembled. Finally, two species without annotations, sugar pine (*Pinus lambertiana*) and axolotl (*Ambystoma mexicanum*), were added to test the upper limits of the methods studied in terms of genome length and sequencing effort. These two species are known for their large genome sizes (27 and 32 GB, respectively) due to the extent of their repeat content (79% and 65.6% are transposable elements; Nowoshilow et al., 2018; Stevens et al., 2016).

Genomes, transcriptomes, and GFF3 annotations of *D. rerio* and *H. sapiens* were downloaded from Ensembl (release 91, assemblies GRCz10 and GRCh38, respectively; Kersey et al., 2018). Assembled genomes, transcriptomes, and annotations from *S. salar* (assembly GCA_000233375.4) and *C. harengus* (assembly GCA_000966335.1) were downloaded from NCBI Genome. Finally, in the case of *A. mexicanum* (assembly GCA_002915635.2) and *P. lambertiana* (GCA_001447015.2 assembly), assemblies were also downloaded from NCBI Genome, while their assembled transcriptomes were taken from the European Nucleotide Archive (accession numbers GFZP01, Nowoshilow et al., 2018; and GEUZ01, Gonzalez‐Ibeas et al., 2016, respectively).

With respect to humans and zebrafish, WGS reads were simulated with wgsim (Li, 2018), while for the other species, they were downloaded from different studies (Atlantic salmon: Kijas et al. (2018); Atlantic herring: Lamichhaney et al. (2012); Ambystoma mexicanum: Keinath et al. (2015); and Pinus lambertiana: Neale et al. (2014); full accession numbers available in Table S1). These assemblies varied in terms of both sequencing depth and individuals, from a 22× of a single individual in axolotl to 51× of a pool of 50 Atlantic herring samples.

### 2.3 Benchmarking metrics

The performance of EXFI was compared with two tools: GMAP (Wu & Watanabe, 2005) and ChopStitch (Khan et al., 2018). GMAP is a method used to perform gapped alignments of expressed sequence tags (ESTs) and assembled transcripts to a reference genome. Its main advantage is that is easy to use and has been used extensively to annotate eukaryotic genomes with PASA (Haas et al., 2003). ChopStitch is a tool similar to EXFI that uses Bloom filters to predict exons and the splice graph, using the entire WGS dataset, and different exon prediction algorithms. Table 2 shows the main differences, advantages, and disadvantages between the three methods.

**TABLE 2:** Qualitative comparison between the three methods studied: GMAP, ChopStitch, and EXFI

Software	GMAP	ChopStitch	EXFI
Input	Genome assembly (FASTA) Transcriptome (FASTA)	WGS reads (FASTQ) Transcriptome (FASTA)	WGS reads (FASTQ) Transcriptome (FASTA)
Output	Alignments (SAM, GFF3)	Exons (FASTA) Splice graph (DOT)	Splice graph (GFA) Exons (FASTA) Gapped transcripts (FASTA)
Steps	Genome index construction Spliced alignment Microexon identification	*k*‐mer cardinality estimation Bloom filter construction Exon prediction Error correction Short exon prediction Splice graph construction	*k*‐mer filtering Bloom filter construction Exon prediction Splice site polishing
Conda?	Yes	No, but via Brew	No, but via Dockerfile
Usability	Easy: index and predict	Easy: build and predict	Easy: build and predict
Genome input	Assembly	WGS	WGS
Sample variability	Genome and transcriptome may come from different sources	Transcriptome and WGS must come from the same individual	WGS reads can come from a Pool‐Seq approach
Large genomes?	Yes (gmapl)	No	Yes
Speed	Fastest (minutes)	Medium (hours)	Slowest (hours)
Memory footprint	Medium/high	Medium/high	Low, adaptable
Precision/recall	Lowest	High	Highest
Mappability	High	High	Highest
Memory–FPR trade‐off	—	Provide FPRs, then reserve optimal memory (may not be available)	Reserve memory, then return the FPR (may be too high).
Main advantage	Popular: easy to install	Fastest genome‐free method	Memory and user‐friendly, most accurate
Main disadvantage	Requires a genome assembly	Highest memory usage	Slowest of the methods

To compare the three methods in terms of speed and accuracy, two metrics were studied: one based on the recovery of the available annotation, and another in terms of mappability of the predicted exons to the genome.

For studying the recovery of the available annotation, reference exon coordinates in GFF format were transformed to BED, converting the chromosome‐based coordinates to transcript‐based, taking into account the strand and order of the exons. For example, if a pair of consecutive exons in transcript A in chromosome 1 are 1:1,000–1,100 and 1:1,500–1,600, they become A:0–100 and A:100–200 with respect to the transcriptome. Once converted, reference and predicted coordinates were compared with the BEDTools intersect subcommand, requiring a mutual overlap of at least 95% of the coordinates. With this program, the standard classification metrics are computed: precision (P = TP/(TP + FP), where TP and FP are the true and false positives, respectively), recall (R = TP/(TP + FN), where FN are the false negatives), and *F*_1_ score (the harmonic mean between precision and recall: *F*_1_ = 2PR/(P + R)). These comparisons are provided in the EXFI package via the compare_to_gff3 script.

In the case of mappability measurements, predicted exons were aligned against their genomic reference with BWA MEM (Li, 2013), results were stored in BAM format with SAMTools (Li et al., 2009), and the reported statistics were obtained from the number of mapped exons, and the ones mapped with a perfect CIGAR string (all matches or with small insertions and deletions, but no base clipping).

### 2.4 Objectives of the benchmarks

Before comparing the three methods, it was necessary to measure the influence of four parameters that may impact performance, in terms of both time and memory consumed, and the trade‐off between precision and speed. From the two metrics described above, we used the annotation‐based statistics as the ones that drove the experimental design since they showed more differences in terms of percentage points, and because mapping methods require a minimum seed length, which impacts the alignment of microexons.

First, the gains in terms of the BF FPR and exon prediction capabilities when reads are filtered or not were studied. Exons form a small fraction of a genome and only WGS reads that overlap the transcriptome are necessary to detect IEBs, while the remainder only increase the memory and BF FPR unnecessarily. The read filtering step implemented in EXFI retains not only exonic reads but also those in the flanking regions, where donor/acceptor signals and small variants can be detected. Therefore, EXFI was applied with and without the read filtering step, fixing the *k*‐mer length to 25 bp, to measure (a) how much it accelerates or slows down the pipeline, (b) the BF FPRs, and (c) the fitness of the predicted exons.

Second, the effects of memory usage were compared. Next‐generation sequencing projects are usually executed in high‐performance computing environments, where RAM memory exceeds orders of magnitude what can be found in desktop and laptop computers. Probabilistic data structures such as Bloom filters have promised great savings in terms of memory, and therefore enabling analyses outside a computing cluster. To explore accuracy under different memory settings, EXFI was executed using the zebrafish dataset multiple times by varying the size of the Bloom filters from 4 to 60 GB in steps of 4 GB, and fixing with the *k*‐mer length to 25 base pairs.

Third, the trade‐off in terms of precision and recall with varying BF *k*‐mer lengths was analyzed. If *k*is set too low, *k*‐mers become less specific and more reads are inserted into the filter, increasing the BF FPR and lowering the precision, while increasing runtime too since there are more k‐mers and reads processed. On the contrary, if *k* is set too high there will be fewer elements to insert, and since a significant fraction of them will contain variants and sequencing errors, they will be filtered by their low frequency (lowering the BF FPR but also the recall). To find the appropriate *k*‐mer length, EXFI was run with the lowest and highest memory settings (4 and 60 GB) and by varying the *k*‐mer length from 21 to 65 using odd values.

Finally, an acceptable genome coverage is needed for a successful experiment. On the one hand, a WGS experiment with little coverage will make the method underperform. On the other hand, too much coverage will make the BF FPR larger than necessary because of sequencing errors. As depth increases, the total number of true *k*‐mers reaches a plateau, while the number of *k*‐mers that contain sequencing errors keeps growing linearly (see figure 3 in Melsted & Pritchard, 2011). Therefore, a central point must exist in between to achieve near‐optimal exon precision and recall values. The zebrafish datasets were sampled in 10% increments with Seqtk (Li, 2018), applying the procedure to each subsample, and measured the classification metrics, using both low and high memory settings and *k* fixed to 25 bp.

With respect to the other tools, GMAP version 2018.07.04 was executed using default parameters, and ChopStitch version 1.0.0, using the default *k*‐mer length (50 bp) when possible, and varying the BF FPR values (and therefore different memory usages), over the six datasets (zebrafish, humans, and Atlantic herring), and we measured the performance in terms of the metrics described above: comparison against the annotations and mapping against the genome. All programs were run on a 2× Intel Xeon E5‐2620 server, running in total 24 2 GHz threads, with 64 GB of RAM.

### 2.5 Retrospective analysis of IEB prediction in *Tinca tinca*

To further validate EXFI for downstream analysis, the method was applied to retrieve the set of 96 transcriptomic SNPs in tench (*T. tinca*) wherein an earlier study (Kumar et al., 2019) was explored, where 92 of 96 were successfully genotyped. EXFI was executed using the assembled tench transcriptome, and the raw genomic reads comprised of two pools of five diploid individuals each, with an overall genome coverage of 52×. Finally, raw reads were mapped to the predicted exons with Bowtie2 (Langmead & Salzberg, 2012), and we performed SNP calling with BCFTools (Li et al., 2009). To derive the genotypable regions of the exons, variants with a quality value below 20 were filtered out, and then those that were within 35 bp to another variant or a predicted exon boundary.

## 3 RESULTS

### 3.1 Human and zebrafish simulations

As a practical approach, for each species a single Illumina HiSeq 2000 run per individual was simulated (360M PE reads), creating WGS datasets with coverages of 54× (2 runs, 720M PE reads) and 70× (6 runs, 2.21B PE reads; Table 1).

### 3.2 Effects of read filtering

**FIGURE 3:**
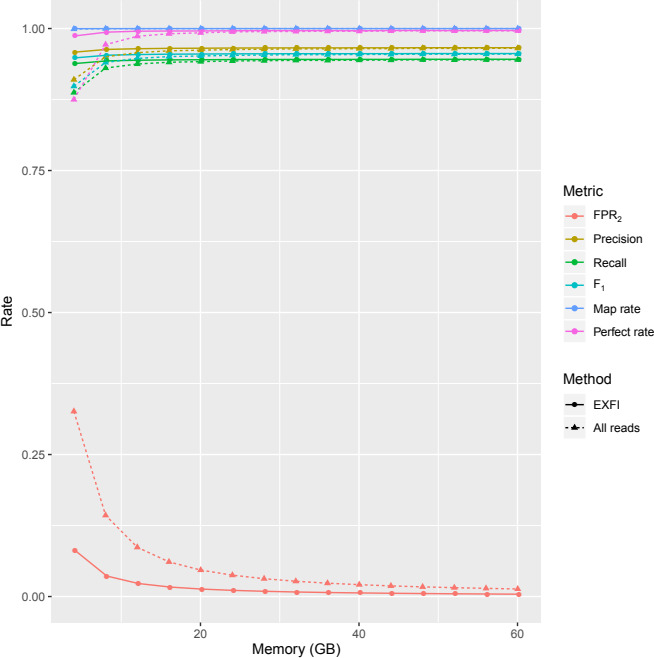
Classification and mapping rates of EXFI depending on Bloom filter sizes in the zebrafish dataset. Filtering the dataset yields better classification and mapping rates by lowering the FPR_2_. These values are already near‐optimal when four Gigabytes are allocated. The raw mapping rates were close to 100% from the start for both methodologies. For the perfect mapping rates, we see EXFI achieving a 98.75% mapping rate from the start

Filtering the reads resulted in a 68%–75% reduction of the BF FPR while also slightly improving all the classification metrics (Figure 3 and Table S2). We can observe a benefit of the filtering in the low memory case, where the FPR fell from 32.6% to 8.1% and rose the *F*_1_ score from 89.8% to 94.8 when the maximum is of 95.6%. Additionally, we observe a slight reduction in time: from 172–186 to 149–173 min (Table S8). Therefore, filtering improves both the processing time and the prediction metrics. Similar conclusions can be reached in the human dataset (Table S3 and Figure S1).

### 3.3 Effects of memory usage

The most significant parameter that impacts the Bloom filter is its size. Figure 3 and Table S2 show the expected decrease in BF FPR as space grew, but surprisingly, the exon precision and recall increased very slowly. Concretely, the BF FPR varied from 8.1% to 0.4% as the memory increased, achieving a 95.8% precision and 93.8% recall in the low memory case, when in the high memory case one both values were respectively 96.6% and 94.6% (Table S2). With respect to the human dataset, experiments were only performed with the low and high memory settings, obtaining BF FPRs of 13.7% and 0.7%, achieving 93.1% and 94.7% precision, and 89.3% and 90.9% recall, respectively (Table S3 and Figure S1). Therefore, a 4 GB Bloom filter is enough to achieve near‐optimal results. Also, it is not necessary to demand particularly low (5% or less) BF FPR to predict exons accurately.

### 3.4 Effects of *k*‐mer length

**FIGURE 4:**
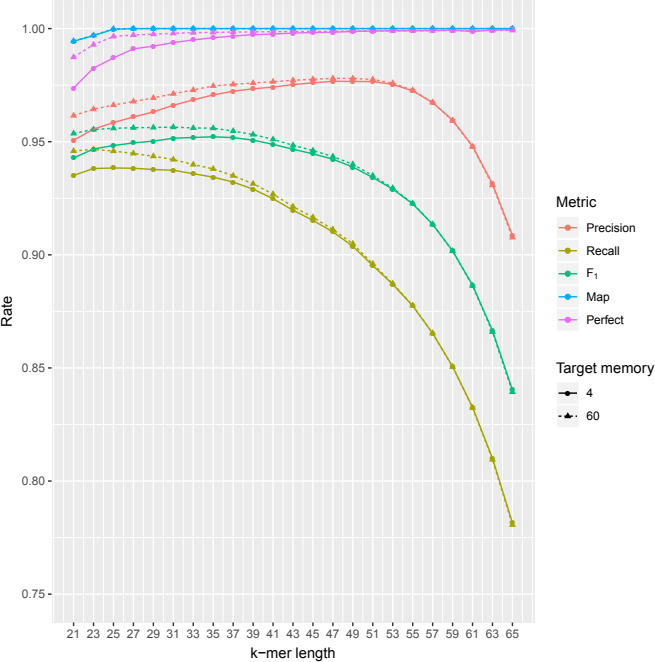
Precision and recall of EXFI when the *k*‐mer length varies, using the minimum and maximum memory settings. As expected, the longer the *k*, the higher the precision, and the lower the recall. For both methods, best results are achieved when the *k*‐mer length varies between 23 and 35. Perfect mapping rates ranged from 97.37% to 99.95%

As described in Methods sections, precision and recall are related through the *k*‐mer length. On the one hand, as *k* increases, the precision also increases until *k* = 47, when it starts to decrease rapidly (Figure 4 and Table S4). On the other hand, recall decreased almost from the start (4 GB: *k* = 25; 60 GB: *k* = 23). According to the *F*_1_ statistic (the harmonic mean between precision and recall), for both methods, there is a window of *k*‐mers, from 23 to 35, where this metric remains stable, boosting the recall when *k* is small, and the precision when k is high. Given the results, we used for the remainder of the analysis a *k*‐mer length of 25 bp to keep the recall as high as possible while keeping precision high too, and set it as the default value in EXFI.

### 3.5 Effects of sequencing depth

**FIGURE 5:**
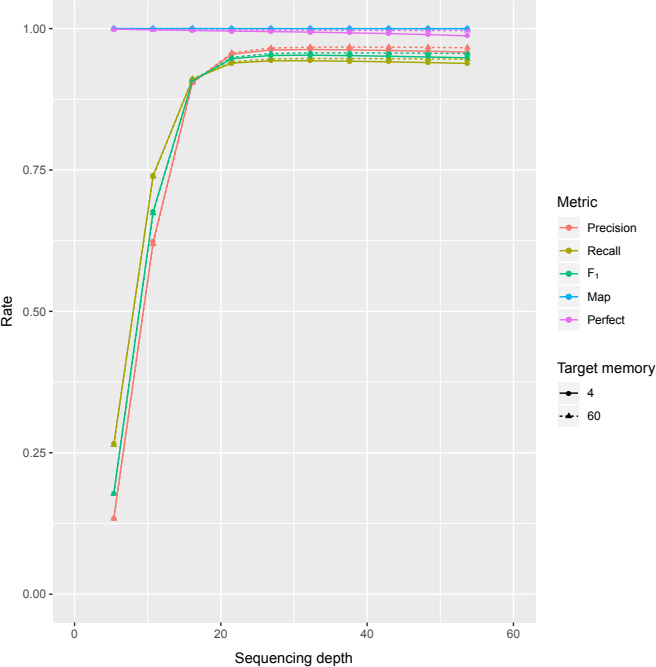
Precision and recall values of EXFI depending on the sequencing depth, using the minimum and maximum memory settings and the *k*‐mer length fixed to 25. Both settings produced similar results, obtaining higher metrics when all the memory was used. Around 25–30× almost all error‐free *k*‐mers are sampled, and then, sequencing errors start to pollute the Bloom filter. Both mapping rates stayed above 98.7%

The sequencing depth increased the power of the precision up to a certain point (Figure 5 and Table S5). For a 16× sequencing depth, precision and recall already are above 90%, and maximum values (96.7% and 94.7%, respectively) are reached when coverage is between 26× and 37×. Past that coverage window, precision and recall start decreasing due to sequencing errors, and unnecessarily raising the BF FPR. Therefore, a sequencing depth of at least 20× is good enough, and that optimally should be between 30× and 40× to retrieve exons from a transcriptome with EXFI.

### 3.6 Comparison with ChopStitch and GMAP on simulated and real datasets

The performance of ChopStitch, EXFI, and GMAP was compared across six species in terms of the BF FPR and sizes, classification, and mappability scores. Given the results above, we chose to run EXFI using 4 GB of RAM, and a *k*‐mer length of 25. For ChopStitch, we used the default *k*‐mer length of 50 bp, and default BF FPRs of 1% when possible. For GMAP, the default parameters were the ones used. In the case of the megagenomes, gmapl was used as the alignment tool.

There are several differences between EXFI and ChopStitch. Algorithmically, in EXFI the total amount of memory to be used is specified at the beginning, the number of hash functions is fixed (to four, fixed number in the version of ABySS used), the reads are filtered and processed, and the BF FPR is returned at the end. In contrast, the reverse procedure is applied in ChopStitch: The desired BF FPRs are first specified, and the optimal sizes and number of hash functions are estimated from the full dataset of reads. This procedure selects the optimal memory (maybe unavailable) and number of hash functions to work, but requires to process twice the full WGS reads: one for estimation and other for actual computations. On the other side, EXFI hashes all the WGS reads in two steps: once for the filtering purpose, and a second time for the remainder.

**FIGURE 6:**
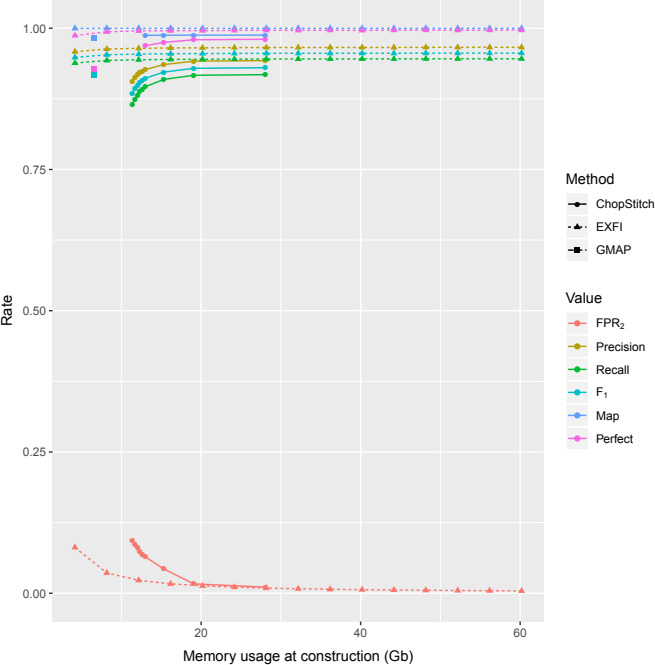
Comparative on memory and precision/recall metrics between ChopStitch, EXFI, and GMAP. EXFI's performance remained high and constant from the start

In zebrafish, we considered running EXFI and ChopStitch with multiple memory/BF FPR configurations (respectively 4–60 GB in 4 GB increments, and FPR_1_ varying from 20% to 1% and BF FPR_2_ set to 1%). In general, EXFI outperformed both methods (Figure 6 and Table S6) and its performance remained high and constant from 4 GB.

When comparing EXFI's low memory mode against ChopStitch default 1% FPR_2_ (and 28 GB) and GMAP (Table 3), we observe that with a BF FPR_2_ of 8% (and 4 GB), EXFI obtained a slightly better *F*_1_ score (with better precision and worse recall) than the other two methods. According to the exon mappability, more than 98% of the predictions of both reference‐free methods were perfectly matched to the genome, while the reference‐based tool obtained 92.7%.

**TABLE 3:** Performance of the three tools across different species

Species	Method	Time	Memory	FPR1	FPR2	Precision	Recall	*F*_1_	Mapped	Perfect
Zebrafish	ChopStitch	1 hr 41 min 54 s	28.060	0.010	0.011	0.943	0.918	0.930	0.988	0.980
Zebrafish	EXFI	2 hr 35 min 15 s	**4.177**	0.256	0.081	**0.958**	**0.938**	**0.948**	**1.000**	**0.987**
Zebrafish	GMAP	**40 min 19 s**	6.567	—	—	0.918	0.917	0.917	0.982	0.927
Human being	ChopStitch*	4 hr 28 min 58 s	30.424	0.158	0.100	0.903	0.868	0.886	0.988	**0.969**
Human being	EXFI	6 hr 32 min 49 s	**4.364**	0.361	0.137	**0.931**	**0.893**	**0.912**	**1.000**	0.957
Human being	GMAP	**1 hr 11 min 25 s**	9.301	—	—	0.883	0.884	0.883	0.985	0.907
Herring R.	ChopStitch	49 min 53 s	5.679	0.010	0.011	0.819	0.858	0.838	0.974	0.965
Herring R.	EXFI	1 hr 25 min 6 s	**4.123**	0.064	0.024	0.816	0.866	0.840	**1.000**	**0.995**
Herring R.	GMAP	**19 min 8 s**	4.707	—	—	**0.949**	**0.941**	**0.945**	0.983	0.935
Herring A.	ChopStitch	50 min 2 s	5.705	0.010	0.011	—	—	—	0.972	**0.871**
Herring A.	EXFI	1 hr 32 min 8 s	**4.111**	0.068	0.026	—	—	—	**0.986**	0.823
Herring A.	GMAP	**37 min 20 s**	6.564	—	—	—	—	—	0.921	0.578
Salmon	ChopStitch	2 hr 57 min 38 s	8.657	0.010	0.010	0.883	0.887	0.885	0.985	0.975
Salmon	EXFI	4 hr 49 min 37 s	**4.466**	0.080	0.042	**0.901**	**0.904**	**0.903**	**0.999**	**0.987**
Salmon	GMAP	**1 hr 22 min 15 s**	9.320	—	—	0.809	0.830	0.819	0.979	0.866
Sugar pine	ChopStitch*	—	—	—	—	—	—	—	—	—
Sugar pine	EXFI	2 days 7 hr 38 min 57 s	60.090	0.090	0.031	—	—	—	**0.997**	**0.903**
Sugar pine	GMAP	**6 hr 20 min 13 s**	**55.371**	—	—	—	—	—	0.956	0.673
Axolotl	ChopStitch*	**14 hr 29 min 38 s**	**29.629**	0.202	0.142	—	—	—	0.851	0.772
Axolotl	EXFI	1 day 3 hr 20 min 50 s	60.313	0.040	0.020	—	—	—	**0.988**	**0.782**
Axolotl	GMAP	—	—	—	—	—	—	—	—	—

Note

Best metrics across the three methods are marked in bold. Time is the sum of the walltimes at the building and prediction steps. When possible, the steps were run using all processors available, that is, in ChopStitch's and EXFI's build steps, and in GMAP's predict stage. Memory, expressed in Gigabytes, represents the peak usage in memory. FPR represents the false‐positive rate of the Bloom filter used for prediction. Mapped and Perfect stands for the overall alignment rate of the predicted exons, allowing and not allowing clipping, respectively. EXFI was executed to use only 4 GB of RAM except for the megagenomes. ChopStitch with *k*‐mer lengths of 50 bp and FPRs of 1%, except when memory usage was an issue. In the cases marked with asterisks, the *k*‐mer lengths were lowered to 25 bp, and target FPR values were tested one by one in the set of 1%, 5%, 10%, 15%, and 20%. Actual FPRs are the ones reported. In general, when a reference transcriptome was used, EXFI obtained the best precision, while ChopStitch obtained better recall. With respect to alignments to the genomes, EXFI obtained the best mapping rates.

With respect to the human dataset, all methods obtained lower metrics than in the zebrafish case, due both to the higher complexity of the transcriptome and the length of the genome. With the default settings, EXFI outperformed both methods with an exon *F*_1_ score of 91.2%. Due to the number of different *k*‐mers to process, ChopStitch's default *k*‐mer length value had to be lowered to 25 and the target BF FPR_1_ had to be raised to 15% in order to avoid memory allocation errors. In this case, ChopStitch obtained an *F*_1_ of 88.6%, and GMAP of 88.3% (Table 3, Table S7, and Figure S2).

In both datasets, GMAP obtained the fastest data structure construction (24 and 53 min to index the zebrafish and human genomes; Table S8), followed by ChopStitch (2 hr 38 min and 4 hr 22 min) and EXFI (2 hr 29 min and 6 hr 18 min). On the other hand, GMAP finished last when predicting (more than 15 min in both cases) and using 24 threads, while for a single compute thread, ChopStitch was the fastest (3 min 41 s and 7 min 18 s in zebrafish and human cases) followed by EXFI (5 min 56 s and 14 min 30 s).

Similar results were obtained when analyzing the salmon transcriptome (Table 3): EXFI obtained the lightest RAM consumption with the cost of obtaining a higher BF FPR_2_ (4.23%), while ChopStitch achieved a 1% BF FPR_2_ with 8.7 GB of RAM. With respect to the prediction of exons, EXFI obtained higher classification and mapping scores, followed by ChopStitch and GMAP.

In the Atlantic herring reference dataset, we observe that both *k*‐mer‐based methods obtained worse‐than‐expected *F*_1_ scores than GMAP when analyzing the reference transcriptome, while still obtaining the highest perfect mappings (in EXFI's case, the highest across all datasets, 99.5%). In the de novo transcriptome case, predictions of all three methods had lower mapping rates than the reference case, with ChopStitch leading the comparison with 87.1% of perfectly mapped transcripts, followed by EXFI (82.3%) and GMAP (57.8%).

Finally, for the axolotl and sugar pine megagenomes, we did not obtain results for all of the methods. Due to the terabase pairs sequenced and the size of the references, ChopStitch was only able to produce a Bloom filter for the axolotl, with a BF FPR_2_ of 20.2% and a *k*‐mer length of 25 bp, and GMAP was able to build both references but failed to produce predictions in the axolotl case due to memory exhaustion. For EXFI, even though it can produce Bloom filters with 4 GB of RAM, the BF FPR_2_s were too high to work (52% and 29.4%, respectively; data not shown), and therefore, we raised the RAM to 60 GB to obtain reasonable FPRs. Indeed, we obtained data structures with BF FPRs of 3.1% and 2.0% in the Pine and axolotl cases and after 2 and 1 days of execution, respectively. In the sugar pine case, 90.3% of the exons were perfectly mapped to the reference (99.7% when clippings were allowed), while GMAP obtained lower results (95.7% mappable, 67.3% without trimming alignments). With respect to axolotl, 78.2% of EXFI's predictions were matched end to end to the genome (and 98.8% at least in part), while ChopStitch obtained a 77.2% rate (85.1% when clipping was allowed).

### 3.7 Retrospective analysis in *T. tinca*

From the set of 266,578 input transcripts, EXFI predicted 1,072,772 exons. In total, after quality and distance filtering, 228,931 SNPs and 26,169 indels were predicted suitable for genotyping. All IEBs proximal to the 96 SNPs described in Kumar et al. (2019) using the Conklin et al. (2013) method were detected by EXFI (therefore 100% precision over this set of SNPs). One SNP was proximal to a false‐positive EXFI IEB, due to multiple variants in a short space, indicating that it would not have been selected for genotyping primer design. Therefore, on this retrospective SNP discovery task, EXFI would recall 95 of 96 of the selected SNPs.

## 4 DISCUSSION

We developed EXFI, a method that reliably predicts the exon sequences and splice graph of a species using a de novo‐assembled transcriptome and raw WGS reads. We tested it in multiple eukaryotic species, varying the genome and transcriptome reference status, simulated and experimental datasets, and samples with different level of heterozygosity of the samples. We found out that EXFI performs better in terms of memory and classification than other tools when describing the structural annotation of every transcript.

We studied the four principal parameters that can affect the prediction procedure: read filtering, memory, *k*‐mer length, and genome coverage. First, by filtering the transcriptome, we ended up reducing by two‐thirds the BF FPR while also slightly decreasing the execution time. Therefore, this reduction can be translated into a memory optimization. Second, using more than 4 GB of RAM (and higher BF FPR) yielded equally accurate predictions as using 60 GB (Figures 3 and 6). Thus, commodity desktop and laptop computers are enough to achieve accurate exon predictions on gigabase‐sized genomes. Third, our approximation predicted a window of optimal *k*‐mer length values between 23 and 35 base pairs. Finally, we show that 20× coverage is good enough for exon prediction, with optimal coverage between 30× and 40×.

We compared EXFI against ChopStitch, a similar method, and GMAP, a splice‐aware program designed to align transcripts to a reference genome. We used datasets that vary in genome size, sequencing depth, number of individuals, and type of input transcriptome. When taking into account the general picture across the different reference species (zebrafish, humans, salmon, and herring), even with higher BF FPR rates, EXFI obtained better prediction metrics, except for the Atlantic herring (Table 3). In that case, EXFI was less accurate predicting exons, but in terms of mappability, it achieved the highest results across all datasets (99.5%). This high mappability result, although more optimistic than the exon precision, means that even if the exon prediction is not precise, it is still usable for downstream analysis. These perfectly matched predictions are interior sections of the exons rather than the full sequence, which makes them suitable for genotyping, array design, and sequence capture.

We also studied three situations where the input transcriptome was de novo‐assembled from RNA‐Seq reads: Atlantic herring, to compare the differences between reference and assembled transcriptomes, and the megagenomes of axolotl and sugar pine. In terms of mappability, all three methods performed worse than in reference cases due to the inherent complexity of transcriptome assembly. In the herring case, the mappability scores fell for all methods. In the axolotl case, we obtained moderate results for EXFI (78.2% perfect mapping) and ChopStitch (71.4%). Finally, in the sugar pine dataset, EXFI's performance stood high (90.3%), while GMAP did moderately (67.3%). Interestingly, results in herring and salmon suggest that reference‐free method remain accurate even when WGS datasets come from a Pool‐Seq approach. Another lesson learned is that special care has to be taken regarding the input transcriptome. While the axolotl and sugar pine transcriptomes come from a wide variety of tissues and conditions and are sequenced in‐depth, the herring transcriptome was obtained from a single tissue, where its characteristic transcripts were assembled in full length, but where the lowly expressed ones appear fragmented, and specific transcripts to other tissues are missing.

Finally, this paper also studied the performance of EXFI in an earlier transcriptomic SNP discovery project in tench (Kumar et al., 2019). EXFI was also able to find hundreds of thousands of SNPs across almost a million exons. With respect to the set of known genotyped exons, EXFI obtained 100% precision and 99% recall.

These positive results for EXFI are due to the read filtering step and the exon prediction rules used. The filtering step is critical in eukaryotic genomes because a significant fraction of the WGS dataset is not only unnecessary but misleading. The convenience of the exon prediction rules is extracted from Figures 3 and 6: When comparing ChopStitch and EXFI without read filtering, the latter obtained slightly superior precision and recall due to the exon prediction methods, in spite of the relatively high BF FPR (8% vs. 1%). Moreover, EXFI's predictions are accurate enough when working with relatively high BF FPR_2_.

Previous structural annotation algorithms rely on a whole‐genome assembly followed by the mapping of RNA‐Seq reads, ESTs and transcripts, and homology predictions against genome, transcriptome, and protein databases. Our results suggest that EXFI is a reliable tool too while avoiding completely the step of generating a high‐quality genome assembly.

Recent reviews have been published on RAD‐Seq and Targeted Sequencing approaches (Harvey, Smith, Glenn, Faircloth, & Brumfield, 2016; Lowry et al., 2017; Meek & Larson, 2019) explaining the advantages and disadvantages of all methods, with the same conclusion: Targeted approaches should be preferred for large quantities of samples and loci. These methods have in common the enrichment of ultraconserved elements (UCEs; Faircloth et al., 2012) or exons under varying selection types. EXFI can be used for both approaches: Conservation of exons can be measured by orthology analysis against other exon predictions and known reference genomes, transcriptomes, and proteomes; and the different selective pressures can be obtained by performing variant calling on the exome given the set of WGS reads used in the analysis.

For optimal results, we propose a two‐step experimental approach to study nonmodel exomes: an initial exploration of the exome structure and the variants it contains, followed by targeted sequencing of hundreds to thousands of samples. For the first step, it would be necessary to sequence RNA from as many tissues and development stages, aiming to get the best representation of the transcriptome, and to sequence between 30× and 40× of the genome, preferably from multiple individuals, to discover as many variants as possible. In this regard, Therkildsen and Palumbi (2017) have shown that is possible to move from pools of DNA to individually barcoded individuals. In a second step, a targeted approach would be obtained for thousands of loci and samples, leaving behind most of the genome and therefore being able to fit more individuals and populations in the same sequencing assay. As it happened for Atlantic herring, a DNA sequencing effort initially focused on the transcriptome (Lamichhaney et al., 2012) was reused years later once genome assembly was possible (Barrio et al., 2016).

This report has presented EXFI, a pipeline that predicts the splice graph and exon sequences from a transcriptome and WGS reads instead of a reference genome. Different parameters that affect its performance were studied: read filtering, memory usage, *k*‐mer length, and sequencing depth. Tests were carried out on zebrafish and human simulations, Pool‐Seq samples of Atlantic salmon and Atlantic herring, and the megagenomes of the sugar pine and axolotl, varying all in sequencing depth, heterozygosity, genome length, and complexity. A retrospective analysis of a recently published set of transcriptomic SNPs on tench was also done, obtaining 100% precision and 99% recall. It is shown that it is possible to perform structural annotation of a transcriptome of heterogeneous samples with low computational resources. Finally, EXFI is expected to be particularly useful for population genetic studies, phylogenetic relationships, and RNA expression in nonmodel species.

## CONFLICT OF INTEREST

The authors declare no conflict of interest.

## AUTHOR CONTRIBUTIONS

**Jorge Langa:** Conceptualization (equal); software (lead); validation (lead); visualization (lead); writing – original draft (equal); writing – review and editing (equal). **Andone Estonba:** Funding acquisition (lead); supervision (equal); writing – original draft (equal); writing – review and editing (equal). **Darrell Conklin:** Conceptualization (equal); funding acquisition (equal); supervision (equal); validation (equal); writing – original draft (equal); writing – review and editing (equal).

## Supporting information

Figure S1

Figure S2

Table S1

Table S2

Table S3

Table S4

Table S5

Table S6

Table S7

Table S8

## Data Availability

EXFI is open source and freely available at https://github.com/jlanga/exfi. It is subject to Continuous Integration and Unit Testing. Detailed installation and usage are available in the README file of the repository (https://github.com/jlanga/exfi/README.md). Additionally, test data are included in the repository. Moreover, a Dockerfile is available at https://github.com/jlanga/exfi‐docker to create a container with all the tools installed. Finally, the scripts to validate, benchmark, and reproduce the tables and figures in this document can be found online as a Snakemake pipeline (Köster & Rahmann, 2012) at https://github.com/jlanga/smsk_exfi_paper. Archived versions of the resources here described are available at Dryad (https://doi.org/10.5061/dryad.tx95x69vc).
