# Supplementary figures and images for: EXFI: Exon and splice graph prediction without a reference genome
Source: Ecol Evol. 2020 Jul 28;10(16):8880–93. doi: 10.1002/ece3.6587 (PMC7452765; doi:10.1002/ece3.6587)

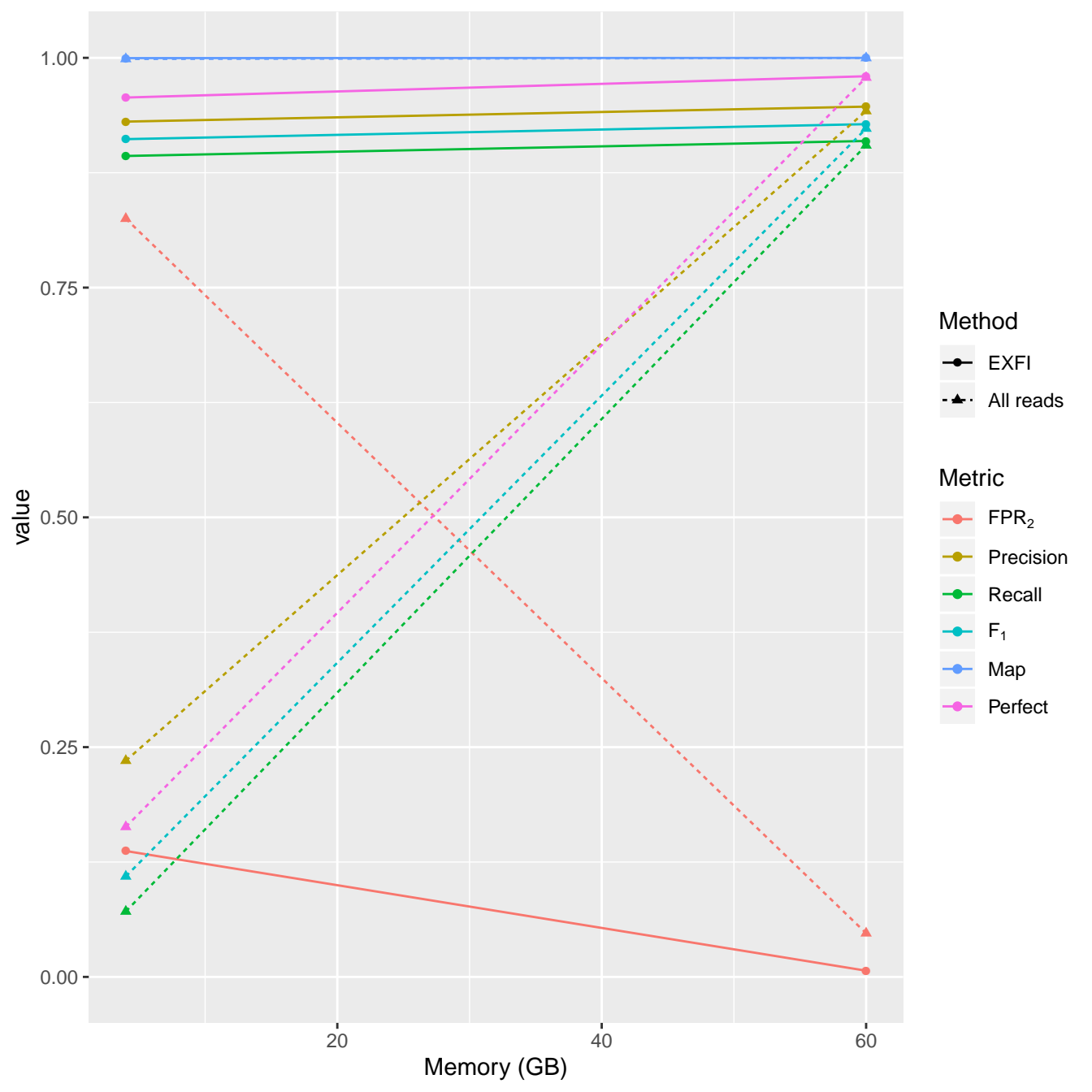

Supplement: Supplementary file 1 — Figure S1 [file ECE3-10-8880-s001.pdf]

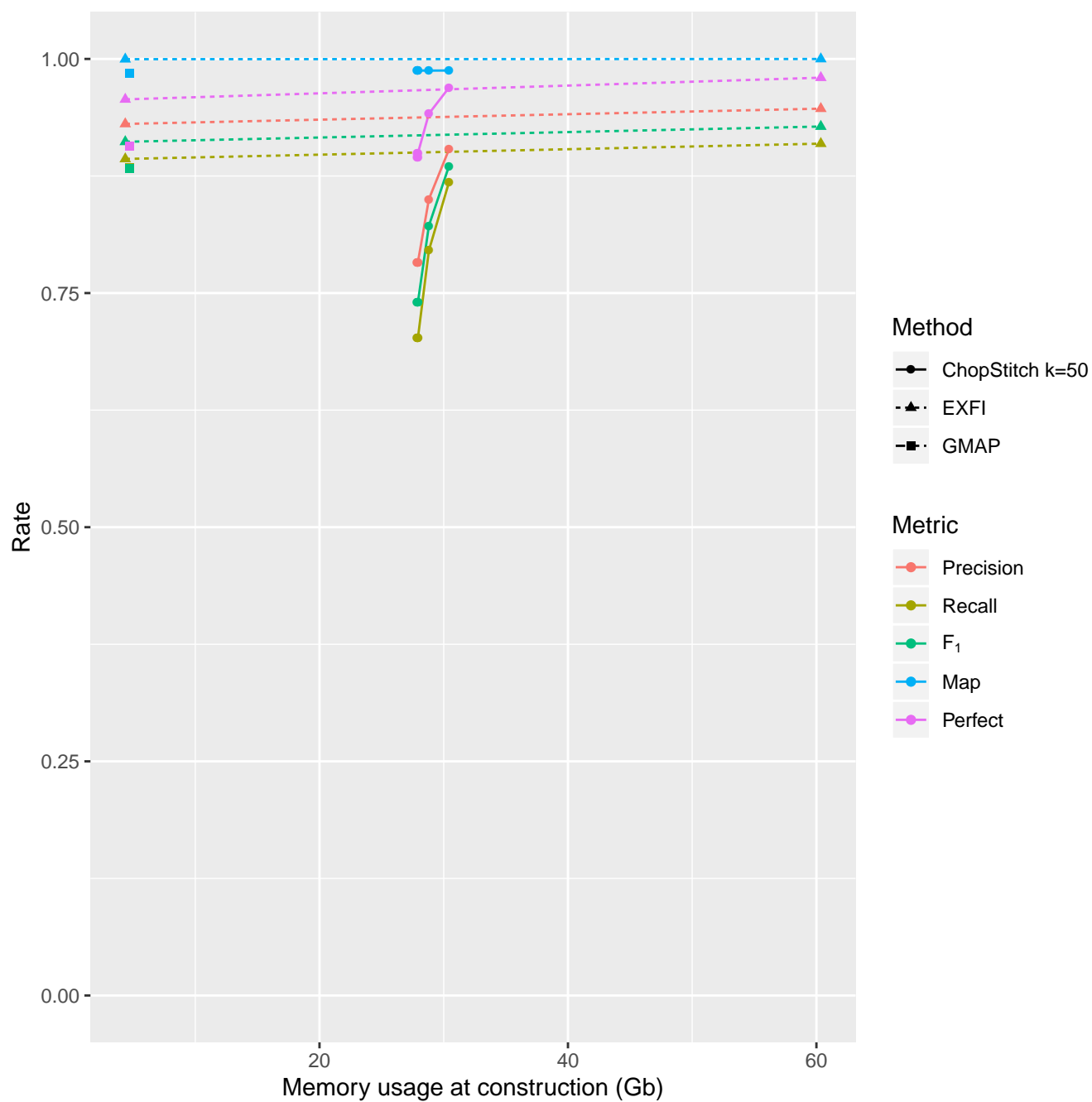

Supplement: Supplementary file 2 — Figure S2 [file ECE3-10-8880-s002.pdf]
